# Prevention of Hospital Associated Venous Thromboembolism in Psychiatric Inpatients- a Survey of Current Practice Within Mental Health Trusts in England

**DOI:** 10.1192/bjo.2022.404

**Published:** 2022-06-20

**Authors:** Ashma Mohamed, Michael Cheah, Josie Jenkinson, Beverley Hunt, Jo Jerrome, Audrey Purcell

**Affiliations:** 1 Surrey and Borders Partnership NHS Foundation Trust, Chertsey, United Kingdom; 2 King's College London, London, United Kingdom; 3 Thrombosis UK, London, United Kingdom; 4 Saint John of God Hospital, Dublin, Ireland

## Abstract

**Aims:**

General hospital inpatients are routinely risk assessed for hospital associated venous thromboembolism (HAT) and given appropriate thromboprophylaxis if indicated. However, mental health trusts have not taken a similar approach in psychiatric inpatients, despite known risk factors, including some unique to psychiatric inpatients. We explored current practice of HAT prevention in English psychiatric inpatients.

**Methods:**

A Freedom of Information Act (FOI) request was sent to all 71 English mental health trusts, asking whether there was a Venous Thromboembolism (VTE) policy, whether a VTE risk assessment tool was being used, what is looked like, and the incidence of HAT in their psychiatric inpatients i.e., VTE during admission or occurring up to 90 days post discharge.

**Results:**

We received 54 unique responses (76%) to the FOI. Of these, 36 (86%) shared their VTE policy, 26 (72%) of which had been adapted for this population; 38 (90%) shared their VTE risk assessment tool, of which 17 (45%) were adapted from the Department of Health VTE risk assessment tool.

Only five trusts out of 42 (12%) monitored VTE events up to 90 days post-discharge and 4 of these shared their monitoring policy. Only 18 (43%) were able to provide data on the number of psychiatric patients diagnosed with a VTE during their stay and up to 90 days post discharge between February 2016–2021, 6 (14%) said they would incur costs to collect this data and 9 (21%) were unable to access this data. Where information was provided, the number of HAT events ranged from 0–224 within each trust. Of the 18 trusts who provided data, a total of 514 events were recorded between Feb 2016-Feb 2021, but none of the trusts were able to confirm if this included VTE events up to 90 days post discharge.

**Conclusion:**

Our FOI survey suggest a high incidence of VTE in psychiatric patients and indicate wide variation in HAT prevention in English hospitalised psychiatric patients. Most had a VTE Trusts had a policy in place, with 45% having a VTE risk assessment tool that listed risk factors unique to psychiatric patients, adapting VTE risk assessment tools in this way may lead to a greater use of thromboprophylaxis. The lack of access to data on HAT by mental health trusts is concerning. Further research is required to understand the rates of VTE, validate a VTE risk assessment tool and conduct trials looking at the benefit of thromboprophylaxis in psychiatric inpatients.

